# Exploring the Leadership–Engagement Nexus: A Moderated Meta-Analysis and Review of Explaining Mechanisms

**DOI:** 10.3390/ijerph18168592

**Published:** 2021-08-14

**Authors:** Anouk Decuypere, Wilmar Schaufeli

**Affiliations:** 1 Research Group HRM and Organisation Behavior, Department of Marketing, Innovation and Organisation, Ghent University, 9000 Ghent, Belgium; 2 Department of Social and Organizational Psychology, Utrecht University, 3584 CS Utrecht, The Netherlands; w.schaufeli@uu.nl; 3 Research Group Work, Organizational and Personnel Psychology, FPPW, KU Leuven, 3000 Leuven, Belgium

**Keywords:** meta-analysis, review, leadership, leadership styles, work engagement, research model

## Abstract

This study aims to review and quantify the value of several well-established positive leadership styles for employee work engagement in organizations. We perform both a quantitative and qualitative review (k = 86). Our (moderated) meta-analysis indicates that transformational, authentic, empowering, ethical, and servant leadership all share overlap in confidence and credibility intervals, and they may result in the same effect on work engagement (general r = 0.47). Additional theoretical analysis indicated a common ground within these positive leadership styles, i.e., having a moral perspective as a leader, role-modelling behaviour, follower self-determination, and positive social exchanges with employees. Based on the studies in the sample, we also build an integrative research model with several categories of mediators and moderators that have a well-established impact on work engagement. The moderator categories were follower characteristics and team- and organizational-level moderators. The mediator categories were psychological needs, trust, resources, and organizational-level variables. The combination of a meta-analysis with systematic review and research model can facilitate future research and supports practitioners to improve leadership.

## 1. Introduction

In these stressful times, it is of crucial importance that leaders support the (psychological) health of their employees. Since today’s organizational environment is characterized by continuous change and renewal [1], day-to-day positive leadership is becoming increasingly important. In a volatile, uncertain, complex, and ambiguous world [2], leaders need to inspire, strengthen, and connect their followers [3]. This will reduce burnout and increase work engagement in organizations [3,4]. Good, visionary leaders provide competitive advantage, especially when firms are facing increasing uncertainty [5], such as with the recent COVID-19 pandemic. Leadership is not only important to envision a firm’s strategy or to decide on an HRM approach at the top of the organization, but also to provide a sense of security and direction for subordinates in every layer of the hierarchy [6]. Even though leadership ‘trickles down’ the organization [7], the immediate supervisor—due to his or her proximal presence and interaction with followers—has a large impact on the day-to-day work environment, performance, and work engagement of employees [8]. This is also shown in Gallup’s work that popularized the idea that employees join companies, but leave bosses. This further underscores the importance of leadership of the immediate supervisor for work engagement and long-term organizational success [9,10]. Arguably, it is the leaders’ responsibility to ensure that conditions are being provided for employees to thrive [3].

Furthermore, thriving (engaged) employees provide a vital competitive advantage for organizations [11], due to the association of work engagement with financial gains for the firm and organizational commitment of employees [12], as well as a service climate, customer loyalty [13], and productivity [14]. A large meta-analysis demonstrates that engagement is also related to health, turnover intentions, and performance [15]. In sum, work engagement has been viewed as one of the most critical drivers of business success [16,17]. Yet, although ‘positive’ leadership styles [18], e.g., transformational, authentic, servant, ethical, and empowering leadership, have been linked to engagement in multiple (longitudinal) studies [19,20,21,22,23], no general framework exists to understand the black box of explaining mechanisms with regards to their effect on engagement.

The development of positive leadership concepts is a fairly recent phenomenon and has been developing over the past 30 years. For instance, the very popular transformational leadership style aims at transforming individual employees’ mindsets toward achieving organizational goals [24]. Other positive [25] leadership styles have been developed and validated as well, e.g., with a stronger focus on normative behaviour [26], on being altruistic as a leader and attuned to the needs and development of employees [27], on being self-aware and authentic [28], or on empowering employees [29]. Examples of other newly developed positive leadership styles are, e.g., shared or distributed leadership [30], benevolent leadership [31], or humble leadership [32].

As a response to this rapid growth in proposed leadership styles, there are calls for an integrative view on leadership [33], for an integration across leadership styles [34], and for an investigation of overlap between leadership styles [35]. This is important to ensure parsimony and make sure that adequate guidelines can be developed for leadership interventions in organizations willing to work in an evidence-based capacity with their leaders. In addition, a synthesis of the field is also important, since several positive leadership styles may not be so different after all with regards to leader behaviours and their effects on performance and wellbeing [18,36]. Therefore, another purpose of this research is to identify the joint mechanisms of positive leadership styles with regards to their effect on work engagement. We want to examine exactly how leaders characterized by different—yet behaviourally not so distinct—leadership styles exert their influence on employee engagement and whether we can bring joint mechanisms together in an overarching research model. To arrive at this ambitious aim, we examine the field both quantitatively, as well as qualitatively.

First, we start with quantitative analyses: we conduct a meta-analysis to establish the magnitude of the association of positive leadership styles in general and for each of the leadership styles separately. Next, we investigate whether the leadership styles in our meta-analysis exert the same influence on engagement through a moderation with leadership style and an investigation of confidence and credibility intervals. We also perform additional moderated meta-analyses with study characteristics. Next, we compare the theoretical underpinnings of positive leadership styles to identify joint mechanisms. Then, the qualitative review continues with systematically analysing the moderators and mediators found in the studies of the meta-analysis. Based on this information, we build an overarching framework to understand the underpinnings of the (positive) leadership–engagement relationship. By bringing these approaches together, we provide a comprehensive quantitative and qualitative review of the up-to-date information with regards to leadership and engagement.

### 1.1. Positive Leadership Styles

We understand positive leadership styles as those leadership styles aimed at having a positive impact on employees [18], as opposed to abusive leadership styles, which have shown to be detrimental for, e.g., employee creativity and wellbeing [37]. In the following section, we will introduce five popular and well-researched positive leadership styles that are also analysed in our meta-analysis and reviewed in the qualitative section.

Transformational leadership is the most popular positive leadership style that has been developed in the past several decades. It focuses on four behavioural dimensions: idealized influence (i.e., leader charisma), intellectual stimulation (i.e., stimulating creativity and innovation), inspirational motivation (i.e., vision provision), and individualized consideration (i.e., considering individual differences) [38]. Therefore, transformational leaders can be described as envisioning a future, acting as a role model, setting performance standards, showing determination and confidence, and being able to transform interactions from ‘pure self-interest to having interest for others’ [39].

Authentic leadership emerged in response of transformational leadership, since scholars suggested differences between authentic and ‘pseudo’ transformational leaders [40,41]. It has been defined as having four components, namely, self-awareness (of the leader), balanced processing (i.e., analysing relevant information before making a decision), relational transparency (i.e., presenting true feelings and thoughts to followers), and internalized moral perspective (i.e., self-regulation based on moral standards and values) [42]. Kernis and Goldman [43] define authenticity as ‘the unobstructed operation of one’s true, or core, self in one’s daily enterprise’ (p. 294), which seems to be related to positive employee outcomes such as work engagement [44].

Servant leadership is characterized by personal integrity and serving others [45]. It is based on the idea that the leader should primarily focus on the needs of others and can be described as an altruistic calling where the focus is on the personal growth of the followers [46,47]. Liden et al. [45] identified seven dimensions of servant leadership, i.e., emotional healing (i.e., showing sensitivity to others’ concerns), creating value for the community (i.e., a genuine concern for helping), conceptual skills (to effectively support and assist others), empowering (i.e., being encouraging and facilitating), helping subordinates grow and succeed (i.e., genuine concern for others’ careers and providing support and mentoring), putting subordinates first (through actions and words), and behaving ethically (i.e., being open, fair, and honest). According to van Dierendonck and Nuijten [48], servant leadership comprises eight dimensions: empowerment (i.e., enabling people and encouraging personal development), accountability (i.e., holding people accountable for performance they can control), standing back (i.e., giving priority to the interest of others first and to give credit to others), humility (i.e., the ability to put one’s own accomplishments and talents in a proper perspective), authenticity (i.e., expressing oneself in ways that are consistent with inner thoughts and feelings), courage (i.e., daring to take risks and trying out new approaches), forgiveness (i.e., when confronted with offenses, arguments, and mistakes), and stewardship (i.e., taking responsibility for the larger institution). In their research, all dimensions, except forgiveness, showed significant correlations with work engagement [48].

In ethical leadership, normative behaviour from the leader is emphasized. Brown et al. [26] defined ethical leadership as ‘the demonstration of normatively appropriate conduct through personal and interpersonal relationships, and the promotion of such conduct to followers through two-way communication, reinforcement, and decision-making’ (p. 120). Ethical leaders are considered to be honest and trustworthy. Brown and Trevino [49] state that ethical leaders distinguish themselves from transformational leaders through emphasizing ethical standards (i.e., being a moral person) and moral management. This moral management can be seen as more transactional, i.e., ‘calling attention to the use of communication and the reward system to send signals about what is important and guide behaviour’ [50].

Empowering leadership is another emerging leadership style that stems from principles based on positive psychology, where there is a focus on enabling employees, rather than enforcing authority [51]. According to Konczak et al. [52], there are six dimensions of leader empowering behaviour: delegation of authority, accountability for outcomes, self-directed decision making, information sharing, skills development, and coaching for innovative performance. In sum, the empowering leader emphasizes the importance of encouraging and enabling followers to lead themselves [53,54].

See Table 1 for an overview of the leadership styles and their components.

### 1.2. Work Engagement

Several conceptualizations and operationalisations of work engagement exist. The most popular and widely used conceptualization is that of Schaufeli and Bakker [55], i.e., engagement is ‘a positive, fulfilling, work-related state of mind that is characterized by vigour, dedication, and absorption’ (p. 295). Vigour is characterized by high levels of energy and mental resilience while working, by the willingness to invest effort in one’s work, and through persistence in the face of difficulties. Dedication is characterized by a sense of significance, enthusiasm, inspiration, pride, and feeling challenge by the task at hand. Lastly, absorption means being fully concentrated and happily engrossed in one’s work, in such a way that time passes quickly and one has difficulties with detaching oneself from work [55,56,57].

An older and slightly different conceptualization can be found in Kahn’s theory on engagement [58]. He explains personal engagement as ‘the harnessing of organization members’ selves to their work roles; in work engagement, people employ and express themselves physically, cognitively, and emotionally during role performances.’ [58]. According to this theory, employees become physically involved, cognitively vigilant, and empathically connected to others through their work. Work engagement can, thus, be seen as a motivational concept whereby employees actively allocate personal resources towards their tasks [11]. The conceptualization of May, Gilson, and Harter [59] is based on the theory of Kahn [58] and comprises three dimensions: the physical component can be described as energy to perform the job, the emotional component refers to ‘putting one’s heart into one’s job’ [60], and the last component, cognitive work engagement, means that one is fully absorbed by their task. Building on Kahn’s work, Rich, Lepine, and Crawford [61] define engagement as ‘the investment of an individual’s complete self into a role’ (p. 617), which is broader than the more popular definition from Schaufeli and colleagues [56].

Macey and Schneider [62], on the other hand, use a broad definition of engagement: they make a distinction between psychological state engagement (i.e., feelings of energy, absorption), behavioural engagement (i.e., extra-role behaviour), and trait engagement (i.e., positive views of life and work). This may help ensure a precision in the definition and conceptualization of employee engagement. In the rest of this article, we will refer to what Macey and Schneider [62] call psychological state engagement, but we will use the more popular term ‘work engagement’ (see e.g., [56]) for clarity, as this seems to be the more popular and accepted term and definition.

### 1.3. Leadership and Engagement: Theoretical Explanations

There are five theoretical explanations for the relationship between positive leadership styles and engagement, i.e., Kahn’s theory for personal engagement, self-determination theory, social exchange theory, social learning theory, and job demands–job resources theory (for an additional overview, see [18]).

First, according to Kahn [58], employee engagement is achieved through fostering three psychological conditions that leaders can impact directly, i.e., psychological meaningfulness, safety, and availability. Psychological meaningfulness refers to a feeling of ‘return on investment’ when someone employs personal energy into their work. It can be enhanced when the leader alters task characteristics (e.g., challenging, varied, creative and autonomous), role characteristics (i.e., do organization members like or dislike the identities and hierarchical stances it requires), and work interactions (e.g., with dignity and a sense of worthwhileness, employing personal and professional elements). Psychological safety can be described as the feeling of being ‘able to show and employ one’s self without fear of negative consequences to self-image, status, or career’ (p. 708). Trust that no harm will come from engagement was related to situations (e.g., predictable, consistent, and clear), interpersonal relations (e.g., supportive, flexible, and open, lower power differences), group dynamics (e.g., voice and hierarchy), the specific management style (supportive, resilient, clarifying, giving autonomy), and clear organizational norms [58]. Psychological availability refers to ‘the sense of having the physical, emotional, or psychological resources to personally engage at a particular moment’ (p. 714). According to Kahn [58], there are four types of distractions from being available for your work: a lack of physical energy or emotional energy, insecurity (based on a lack of self-confidence, self-consciousness, and an ambivalence regarding the fit with the organization and its purpose), and outside life (being too preoccupied). Thus, when a leader provides meaningful work, makes sure there is psychological safety, provides resources that enhance energy, and builds up levels of confidence of an employee, engagement will increase.

Second, self-determination theory (SDT; [63]) posits the importance of psychological needs, which can be influenced by the leader as well. It states that autonomy, i.e., volition and (psychological) freedom, relatedness, i.e., being connected to others, and competence, i.e., feeling effective, are important to reach an autonomous, intrinsic motivation. This has been related to engagement as well [64]. So, when a leader focuses on (1) empowering employees (autonomy), (2) enhancing relationships on the work floor (relatedness), and (3) providing training and feedback to increase levels of competence, work engagement will improve. Engaging leadership also bases itself on psychological need satisfaction [3] and states that those who inspire, strengthen, and connect followers enhance work engagement.

Third, social learning theory posits that leaders can influence positive organizational behaviour (e.g., engagement) through behavioural modelling [65,66]. In this sense, when leaders are engaged themselves, they may serve as role models from which employees may want to emulate the engaged behaviour [18]. Moreover, this process can also be unconscious/emotional, since research on the crossover of burnout and engagement has shown that engagement is also contagious among a group members [67].

Fourth, according to social exchange theory (SET; [68,69], the exchange relationship between supervisor and employee is maintained through a state of interdependence where there is an expectation of reciprocation of favours, work, or support. This means that trust may be a key concept in linking leadership with engagement [70]. Indeed, several empirical studies show that leaders might enhance wellbeing through building trusting relationships [19,71,72].

Fifth, the job demands, job resources theory indicates that both job demands and job resources contribute to work engagement through both a stress process, in which excessive demands have a negative impact, and a motivational process, in which job resources foster work engagement [55]. Since leaders have the capacity to influence job demands and resources, they may indirectly influence work engagement as well [3].

Based on these theoretical considerations and individual studies that show the link between several positive leadership styles and work engagement (for an overview, see, e.g., [18]), we posit the following overarching hypothesis:

**Hypothesis** **1** **(H1).** *All the positive leadership styles in our study associated with work engagement*.

In addition, we posit that these positive leadership styles may also share overlap, theoretically and empirically, with regards to their effect on positive leadership styles:

Theoretically, the notion of a common ground is supported by scholars [35] who state that ‘in general, meaningful similarities exist because each leadership construct was developed for the same purposes, namely, to account for leaders’ behaviours at work and to explain variance in followers’ criteria like motivation or commitment.’ (p. 142).

Empirically, there are also two meta-analyses (with fewer styles and studies than this one) indicating that work engagement is associated with authentic leadership ([73]), as well as with servant leadership, ethical leadership, authentic leadership, and transformational leadership ([66]). In addition, these two meta-analyses showed a high association between several positive leadership styles, i.e., between authentic leadership and transformational leadership ([73]) as well as between ethical, authentic, servant, and transformational leadership ([66]). This is an indication of a common ground, or construct redundancy, between several positive leadership styles, which is also echoed in meta-analytic research concerning leader behaviours [74]. Therefore, we also posit the following hypothesis:

**Hypothesis** **2** **(H2).** *All the positive leadership styles in our study share (theoretical and empirical) overlap with regards to their effect on work engagement*.

To test Hypothesis 1 and the empirical section of Hypothesis 2, we performed a meta-analysis. To further quantify Hypothesis 2, we continue with a theoretical and empirical review of shared mechanisms.

## 2. Materials and Methods

### 2.1. Literature Search

Three comprehensive literature searches were conducted at different time points (2014, 2016, 2018) in all relevant scholarly computerized databases including Web of Science, EBSCO business premier, PsychInfo, Google Scholar, ABI/INFORM, and SocINDEX. Different combinations of key words were used (for the title and abstract), including the terms leader, manager, supervisor, and work engagement as well as employee engagement. The following sequence of key words was entered in the most search engines: (leader* OR manage* OR supervis*) AND (‘work engagement’ OR ‘employee engagement’). In addition, reference lists of relevant or highly cited (review) articles (e.g., [66,73,75,76] and books (e.g., [77]) were scanned in order to identify additional articles.

A three-step screening strategy was used. First, the resulting articles in the search engine were scanned on titles and—if relevant—abstracts as well. Second, the full articles were investigated. Last, when articles did not provide adequate quantitative data, authors were consulted. Scholars that researched leadership and engagement as a focal question in their studies were contacted to ask for more studies. This process was repeated three times to ensure a higher number of studies in each leadership category. Therefore, in the third step of stage 2 and 3, doubles were also omitted. A summary table of the main characteristics of the articles can be consulted in the Appendix A. The articles that were used in the meta-analysis are indicated with an asterisk in the references.

### 2.2. Inclusion Criteria

We included articles that were published in scientific, peer-reviewed journals to make sure the quality of data and analysis was adequate: articles also had to contain validated measures of leadership and engagement. We excluded studies that examined a very specific type of leadership, e.g., benevolent leadership [31], leader identity entrepreneurship [78], and humble leadership [32], since there was not enough empirical research to warrant a separate category in the meta-analysis. We excluded research on leader–member exchange, since this cannot truly be categorized as a leadership style. Rather, it is an exchange mechanism that can be shared across leadership styles (see ‘Shared Themes’ below). In addition, one study [79] studied interpersonal leadership, but used a transformational leadership questionnaire, so we retained that study. We also excluded articles when there was a secondary analysis of a previously included article in our database [80], when the authors could not provide the necessary information (e.g., r), when state rather than trait engagement was measured (e.g., through diary studies, [81]), when work engagement or the perception of leadership was measured at the team level [82,83], or when there was a time lag in the measurement of leadership or engagement (because of the lack of comparability).

Three articles in our dataset provided information regarding two leadership styles, based on the same sample. We decided that only one result would be included, to ensure sample independence [84]. We chose the results from leadership style with the smallest amount of studies in our meta-analysis; this meant servant leadership in one study [47] and authentic leadership in another study [85].

### 2.3. Analyses

The Metafor package for R was used to conduct the meta-analysis [86]. We chose the Pearson r correlation as our effect size, since it was reported in most articles and can be recommended as a good effect size measure [87]. When articles only reported correlations with subscales of the leadership or engagement questionnaire, we calculated averages. This may lead to an underestimation of the true correlation, since the compound construct correlation with a criterion is often larger than an average of the constituent constructs [88]. We followed the meta-analysis method from Hunter and Schmidt [84]. In order to perform a ‘bare bones meta-analysis’ with the Metafor package, we followed three steps: (1) we used an adjusted method for the calculation of the sampling variances, (2) we used the sample sizes as weights, and (3) we used the Hunter and Schmidt estimator for heterogeneity. In addition, we corrected for attenuation through taking into account the reliabilities of the individual studies (see also [84]). When they were not provided, we used an average for the specific measure (see Appendix A). To check the normality assumption of the random-effects model, we investigated a quantile–quantile (q-q) plot, which indicated that a correction for the assumption of a normal distribution was not necessary.

Cochran’s Q-test [89] investigates whether the variability in the observed correlations is larger than would be expected based on the sample variability. A significant test, thus, suggests that the outcomes are heterogeneous [86] due to methodological diversity or the influence of other moderators. We also used this test to determine whether some study characteristics were moderators: we tested for the influence of the industry, western vs. non-western samples, and whether the UWES was used to measured engagement or not. To assess the effect of industry, the studies were divided into nine categories. To test the other effects, we used dummy coding. In order to investigate Hypothesis 2, we also tested whether leadership style moderated the total effect on work engagement.

We provide both the 95% confidence interval and 80% credibility interval around the estimated true population correlation. The confidence interval provides an indication of the precision with which the correlation has been estimated [90]. It can be interpreted in this way: if you were to calculate the estimate of the population correlation multiple times, the true mean would be between the upper and lower bound of the interval in 95% of the cases; we can be 95% confident of the CI estimates. Put differently, the distribution of obtained effect sizes is very unlikely (5%) to fall outside the range specified in the confidence interval. We then evaluate the significance of the correlation estimate by examining whether the associated confidence interval includes 0 or not.

The credibility interval is a Bayesian statistic, which is associated with the (posterior) distribution of the population parameter, since (population) parameters are treated as random variables; the assumption is, thus, that the true population mean of the correlation can take a range of values. The interval indicates where 80% of the true effects are expected to fall [86]; 80% of the time, the true population correlations fall within the range specified in the interval. Since it is a prediction, the outcome is, therefore, 80% credible. In addition, when this interval is large or includes zero, there might be moderators influencing the relationship [91]. With regards to a positive correlation, an 80% credibility interval excluding zero indicates that more than 90% of the individual correlations are greater than zero, since 10% lie beyond the upper bound of the interval [92].

According to Judge and Piccolo [92] ‘confidence intervals estimate variability in the mean correlation, whereas credibility intervals estimate variability in the individual correlations across the studies.’ (p. 758). The intervals also provide information with regards to the comparison of the correlation coefficients: if the intervals do not overlap, it suggests that the subgroups (i.e., the different positive leadership styles) are independent; when they do overlap, it suggests that they might result in the same effects on engagement, with a likelihood of 95 and 80 percent, respectively [84].

According to Rothstein, Sutton, and Borenstein [93] (p. 1): ‘publication bias is the term for what occurs whenever the research that appears in the published literature is systematically unrepresentative of the population of completed studies’. It is based on the assumption that articles are usually only accepted when results are statistically significant. Therefore, a meta-analysis may overestimate the effect size in the true population. To investigate publication bias, we calculated the fail-safe N [94], which results in a metric that shows how many non-significant studies would have to be included in the analysis to change the results to non-significant (0.05 by default). However, the failsafe N is not an optimal means to establish publication bias [95], so we opted for an additional publication bias metric, i.e., the funnel plot and trim and fill analysis.

The funnel plot is one of the most common methods to investigate possible publication bias; it is a graphical representation of the individual effect sizes and standard errors. All meta-analytic analyses reported in the current study were carried out using a method based on the funnel plot: i.e., the non-parametric (rank-based) trim and fill algorithm developed by Duval and Tweedie [96,97]. This is a data augmentation technique that uses the funnel plot to reduce the effect of publication bias; it estimates the missing studies based on the suppression of the most extreme results on one side of the funnel plot and, then, augments the observed data with the goal of making the funnel plot more symmetric, after which it recomputes the estimates [86]. In Table 2, the r indicates the corrected correlation based on this method.

## 3. Results

### 3.1. General Characteristics of Studies

The total amount of studies in the meta-analysis (k = 86) came from samples from 30 different countries. The studies were conducted in both Western (US, Canada, Western-Europe; 48%) and non-Western countries (52%). The total sample also comprised a variety of industries and jobs. We divided them into nine categories: education (12.8%), IT/consulting (4.6%), nursing/hospitals (11.6%), hospitality/service industry (9.3%), finance/banking (10.4%), manufacturing/chemical (6.9%), logistics/maintenance (4.6%), and police/fire fighters (2.3%). Most studies investigated various industries or jobs in the same sample (37.2%). This shows the heterogeneity of the final sample, which supports the generalizability. More details can be found in the Appendix A.

### 3.2. Leadership Questionnaires

With regards to transformational leadership (k = 43), the most frequently used questionnaire (62.8%) was the Multifactor Leadership Questionnaire (MLQ) from Bass and Avolio [98]. With regards to ethical leadership (k = 10), all but one of the studies used the Ethical Leadership Scale from Brown et al. [26]. Servant leadership (k = 4) was measured with three different questionnaires, of which the Servant Leadership Scale [48] was used twice. Authentic leadership (k = 21) was mostly measured (76%) with the Authentic Leadership Questionnaire (ALQ; [42]). Lastly, empowering leadership (k = 8) was measured three times with both the Leader Empowering Behavior Questionnaire (LEBQ; [52]) and the Leader Behavior Questionnaire [52]. The other two studies used the questionnaire from Ahearne et al. [99].

### 3.3. Engagement Questionnaires

Most of the studies (73; 84.9%) used some version of the Utrecht Work Engagement Scale (UWES; [56]), the majority (50; 68%) chose the nine-item version. The Work Engagement Scale from Rich et al. [61] was administered four times. The engagement scale from Saks et al. [100] was used twice, as well as the DDI E3 (as used in Popli and Rizvi [101]). Three studies used questionnaires from Gallup: the Gallup Workplace Audit [102] and the Gallup Q12 Employee Engagement Questionnaire [9], as used by Sahu et al. [103]. Other engagement scales that were used only once included a Work Engagement Scale from Rothmann [104] and 18 items from Watson [105].

### 3.4. General Results of the Meta-Analysis

Table 2 displays the main results of the meta-analysis. According to the classification of Cohen [106], the general correlation between leadership and engagement can be qualified as medium (r = 0.47, *p* < 0.001). Other scholars argue that the cut-off values presented by Cohen [106] may be overestimates for magnitudes of relationships: a more empirical approach for classifying effect sizes shows that the correlations found in our study are rather large [107]. In addition, according to Hemphill [108], the associations found in the meta-analysis are rather strong in comparison with other meta-analytic psychology research. Regardless, 22.09% of the variance in work engagement can be explained by the positive leadership styles in the sample. We also performed meta-analyses on each separate positive leadership style. All of them showed significant medium to large correlations with work engagement (see Table 2). The variance explained ranged from 9.61% (empowering leadership) to 31.36% (ethical leadership).

With regards to publication bias, the fail-safe N indicated that a rather large number of other study results (i.e., 254,154 for the total effect) would be necessary to make the outcomes of the meta-analysis non-significant. Furthermore, the results for most analyses remained the same with or without the trim and fill method [96,97]. Only for the servant leadership (k = 4), the correlation was lessened, and for empowering leadership (k = 8), the correlation was augmented. These results may be due to the smaller sample sizes.

The results for each subgroup of leadership styles have overlapping credibility and confidence intervals, suggesting that they may have the same effect on work engagement. In addition, a combination of the Q-test and some of the (wider) credibility intervals indicate that there might be significant heterogeneity or variation between the studies, which indicates the necessity of a moderation analysis.

### 3.5. Additional Analyses: Moderated Meta-Analysis

First, we tested whether leadership style moderated the total leadership effect on engagement. This effect was not significant [QM(4) = 4.53, *p* > 0.05], further supporting Hypothesis 2. In addition, the general effect of industry was also not significant [QM(8) = 11.32, *p* > 0.05], although the individual factor results did indicate that the correlation in the education category was lower (correlation difference (∆r) = −0.16, *p* < 0.05). The moderation with regards to the engagement questionnaire (UWES vs. non UWES) was not significant [QM(1) = 1.36, *p* > 0.05]. There was also no difference with regards to sample size [QM(1) = 0.0003, *p* > 0.05], nor publication year [QM(1) = 0.53, *p* > 0.05] or western vs. non-western samples [QM(1) = 1.18, *p* > 0.05].

We also tested the effects of the leadership questionnaire and engagement questionnaire for each leadership style. With regards to transformational leadership, we found no effect when we compared the Multifaceted Leadership Questionnaire [24,38,98] to other transformational leadership measures [QM(1) = 3.48, *p* > 0.05]. The other questionnaires did have slightly lower correlations, but this effect failed to reach significance (correlation difference (∆r) = −0.12, *p* = 0.06). The effect of the engagement questionnaires was also not significant [QM(1) = 1.23, *p* > 0.05]. With regards to authentic leadership, the difference between the questionnaire based on Walumbwa et al. [42] vs. the others was also not significant [QM(1) = 0.00, *p* > 0.05], as was the moderating effect of the engagement questionnaires [QM(1) = 1.51, *p* > 0.05]. With regards to empowering leadership, the moderating effect of leadership questionnaires was also not significant [QM(2) = 4.47, *p* > 0.05], although the two studies with the questionnaire from Ahearne et al. 99] did show a higher correlation (∆r = 0.15, *p* < 0.05). This was the only leadership style where the kind of engagement questionnaire did have a moderating effect [QM(1) = 5.00, *p* < 0.05], although it is only based on very few studies: the two studies that did not use the UWES had a higher correlation with engagement (∆r = 0.14, *p* < 0.05).

The amount of studies (k = 4) and different leadership questionnaires (3) with regards to servant leadership made the moderation effect not relevant to test. In addition, all the studies used an UWES variant to measure engagement. With regards to ethical leadership, all studies but one were measured with the questionnaire from Brown Treviño and Harrison [26], and all but one used UWES to measure engagement, which is why this leadership style was also not further explored with regards to the moderating effect of the style of leadership or engagement questionnaire.

### 3.6. Conclusion

All positive leadership styles, including empowering leadership, were significantly and positively related to work engagement. In addition, all CI and CR intervals showed overlap, indicating that these positive leadership styles partly result in the same effect on work engagement. This supports Hypotheses 1 and 2 and warrants a deep dive into shared mechanisms (see below). Furthermore, education level of employees, leadership or engagement questionnaire, sample size, and publication year did not moderate the relationship between leadership and engagement.

## 4. Theoretical Analysis: The Core of Positive Leader Behaviour

In order to deduce the presence of shared elements with regards to positive leadership styles, we first compare the founding theories of these positive leadership styles. We base ourselves on four elaborate comparative research studies.

First, Gregory Stone et al. [109] wrote that transformational and servant leadership share a focus on influence, *vision*, trust, respect or credibility, risk-sharing or delegation, integrity, and role modelling. They concluded that ‘the theories are probably most similar in their emphasis upon *individualized consideration* and *appreciation of followers*.’ (p. 6). These are relevant behaviours for engagement: vision, e.g., might enhance followers’ meaningfulness of work and, therefore, enhance engagement [110].

Second, according to Walumbwa et al. [42], having an internalized moral perspective (authentic leadership) and being a ‘moral person’ (ethical leadership) were the main shared components. Being a ‘moral manager’ (ethical leadership) was less important in authentic and transformational leadership. Furthermore, ‘idealized influence’ (transformational leadership) was somewhat less pronounced in authentic leadership. Hence, it can be concluded that these four shared attributes are all associated with being a ‘moral’ person or being a ‘moral role model’ as a leader. This is also the case for the facet idealized influence (derived from transformational leadership), which can be described as: ‘role models for followers to emulate; can be counted on to do the right thing; and display high standards of ethical and moral conduct’ [38,42].

Third, Avolio and Gardner [111] compared servant with transformational leadership based on the components of the authentic leadership development theory. A positive moral perspective, leader self-awareness (of values, cognitions, and emotions), positive role-modelling, self-determination, and follower self-awareness of values were all shared focal points. Follower development through supporting self-determination and enhancing follower self-awareness of values [111] can be related to a fundamentally motivational process, where need satisfaction leads to an autonomous motivation [112] as well as to work engagement [47].

Last, Brown and Trevino [49] point out that concern for others (i.e., altruism), ethical decision making, a sense of integrity, and role modelling were shared leadership attributes between transformational, authentic, and ethical leadership.

Empowering leadership. To the best of our knowledge, empowering leadership has not been thoroughly compared with other positive leadership styles. Gregory Stone [109] p. 6 mentioned that ‘empowering followers’ was emphasized in both transformational and servant leadership, indicating overlap between the leadership behaviours in these styles. Empowering leadership can also be related to authentic and transformational leadership, since they focus on the development of employees through fostering follower self-determination [73]. This is also a focal point on servant, transformational, and authentic leadership [76].

### 4.1. Shared Themes

A first recurring theme in the theoretical comparisons of the four positive leadership styles seems to be the focus on a moral perspective and role modelling behaviour (see Table 3). This view is echoed by Avolio and Gardner [111] who posit that authentic leadership, and the focus on morality, is a root concept or precursor to other forms of positive leadership. Role modelling through an internalized perspective and through being a moral person [42,109] enhances the capacity of a leader to be an example for future employee behaviour. The central role of moral development is also substantiated in the work of Day, Harrison, and Halpin [113], see chapter 6 ‘Moral Development’). In this work, the authors elaborate that the moral and ethical development of a leader is important, since (1) every leader needs to be able to make ethical decisions, (2) leaders are role models whose behaviour are emulated by followers, and (3) leaders shape the organizational climate. This explanation also indicates that moral development and role modelling behaviour seem to be intertwined. In addition, the authors found that moral reasoning and development is emphasized in different leadership styles, including transformational, ethical, servant, and authentic leadership 113]. Recent meta-analytic research supports this view and shows that moral and values-based leader behaviours are emphasized in different leadership styles, i.e., authentic, charismatic, ethical, and servant leadership. In addition, these behaviours show strong correlations to critical employee outcomes (e.g., performance, OCB, and turnover intentions; [74]). Other conceptual work on the moral content that undergirds positive leadership styles takes this a step further and argues that even though servant, authentic, and ethical leadership styles share a focus on morality, each of these styles also have ‘a unique and even contrasting answer to the question: “What is moral?”’ [114] (p. 149). The authors propose that servant leadership focuses more on consequentialism and reciprocity, ethical leadership focuses more on standard of behaviour and deontology, and authentic leadership focuses more on moral autonomy and virtue ethics. However, the relevance of morality remains core to these leadership styles and their effectiveness.

A second recurring theme is the importance of positive social exchanges or LMX for different leadership styles. This was shown in the theoretical comparison from Avolio and Gardner [111] concerning the overlap between transformational, servant, and ethical leadership (see Table 3). Several (meta-analytic) studies back up this theoretical claim. First, a meta-analysis that viewed LMX as a leadership style found meaningful correlations with, e.g., transformational leadership [35]. Second, a theory-based meta-analytic study by Ng [115] also highlighted the critical role of LMX in supporting leadership to exert its effects. Third, a recent meta-analysis points out high correlations between these four positive leadership styles and LMX (r = 0.65–0.71; see [66]), showing that they are all related to positive social exchanges with employees. Fourth, recent research utilizing a combination of meta-analysis and structural equation modelling (i.e., MASEM) identified leader–member exchange as the most dominant mediator category in the leadership–performance relationship [36]. Fifth, research on leader behaviours also finds these high correlations between values-based and moral behaviour models with critical outcomes such as LMX [74]. The authors posit the possibility of contamination of leadership constructs with other variables such as LMX. In any case, both theoretical and empirical research seem to indicate a strong relationship and perhaps overlap between positive leadership styles and LMX.

Finally, if we take into account the newly developed empowering leadership and its relationship with other leadership styles, the development of employee self-determination may be shared across positive leadership styles as well [73,76].

### 4.2. Conclusion

These theoretical findings show that there is evidence for overlap in each of the investigated leadership styles. Some of these shared leader behaviours are concerned with having a moral perspective, modelling behaviour, supporting self-determination, and positive exchanges with employees.

## 5. Building the Research Model: Mediating and Moderating Mechanisms

In addition to the shared effect on engagement, positive leadership styles may also work through the same mediating and moderating mechanisms. Therefore, in addition to the quantitative (moderated) meta-analysis above, we continue the qualitative review to determine which moderating and mediating mechanisms are more plausible to have an effect on the association between the five positive leadership styles and engagement. For this purpose, we re-used the studies from the systematic search sample.

### 5.1. Moderating Mechanisms

In total, there were 14 studies with mostly individual-level moderators based on the sample of studies from the meta-analysis. As can be seen in Table 4, high levels of the individual-level moderators positively influenced the effect of leadership on engagement. Of these studies, only promotion focus was found to have an effect twice, both with transformational leadership [6] and ethical leadership [116]. In addition, three organizational level mediators were found: high uncertainty augmented the relationship between servant leadership and engagement [117] and a more supportive culture heightened the relationship between transformational leadership and engagement [118], while beneficiary contact lessened the impact of authentic leadership on engagement [119]. There was only one team-level moderator: group job satisfaction diminished the relationship between ethical leadership and engagement [120]. These studies are too diverse to draw any conclusions with regards to shared moderating variables. Therefore, Hypothesis 2 concerning shared moderating variables in the relationship between positive leadership styles and engagement cannot be confirmed with studies from the systematic review. The heterogeneity with regards to moderators in the positive leadership–engagement relationship does indicate the need for more research with regards to boundary conditions.

### 5.2. Mediating Mechanisms

Of the studies included in the meta-analysis, 51 mediators were found for the relationship between a positive leadership style and engagement. They were organized in several categories, i.e., psychological needs, trust, job and personal resources, organizational level mediators, and other categories.

1. Psychological needs. As can be seen in Table 5, most studies (13) related to psychological needs. First, several studies found psychological needs as conceptualized by self-determination theory [63] to be a mediator in the relationship between positive leadership styles and engagement, i.e., competence need satisfaction [129], relatedness need satisfaction [129], and total psychological need satisfaction [47,125]. Second, four studies investigated work meaningfulness as a mediator. This is not surprising, since Kahn [58] already proposed that psychological meaningfulness, along with availability and safety, were precursors of work engagement. Both Kahn [58] and SDT proposed theories concerning antecedents for engagement (see Section 1), which can be influenced by positive leadership.
  Third, psychological empowerment was found to be a significant mediator in five studies with different positive leadership styles. Since this is a relatively new concept, we will provide the definition: ‘increased intrinsic task motivation manifested in a set of four cognitions reflecting an individual’s orientation to his or her work role: competence, impact, meaning, and self-determination’ [157] (p. 1443). Competence is defined as ‘an individual’s belief in his or her capability to perform activities with skill’ (p. 1443). Having an impact is defined as ‘the degree to which an individual can influence strategic, administrative, or operating outcomes at work’ (p. 1444). The third element, meaning, is defined as ‘the value of a work goal or purpose, judged in relation to an individual’s own ideals or standard’ (p. 1443). Lastly, the self-determination component is defined as ‘an individual’s sense of having choice in initiating and regulating action’ (p. 1443). The definitions hint at meaningfulness, competence, autonomy, as well as full self-determination; therefore, we categorized this concept under the label ‘psychological needs’.
  In sum, these studies indicate that the satisfaction of psychological needs may be the primary mechanism through which positive leadership influences engagement: leadership that enhances the fulfilment of psychological needs (SDT) or psychological conditions [58] enhances work engagement.
2. Trust. Trust in the leader (k = 8) or organization (k = 2) was found to be a mediator in ten different studies. Trust can be defined as ‘a psychological state comprising the intention to accept vulnerability based upon positive expectations of the intentions or behaviours of another’ [158] (p. 395). Trust can be related to engagement in several ways. Macey and Schneider [62] point out that ‘engaged employees invest their energy, time, or personal resources, trusting that the investment will be rewarded (intrinsically or extrinsically) in some meaningful way’ (p. 22). This is similar to what social exchange theory posits (SET [68]; see introduction). In this view, the exchange relationship between the leader and employee is maintained through a state of interdependence: there is an expectation of reciprocation of favours, work, or support based on mutual long-term investment, socio-emotional give-and-take, and trust. Indeed, several other authors see (interpersonal) trust as a part of a quality social exchange relationship [159,160]. This relation-based perspective on trust is, therefore, based on mutual obligation [69,161]. When employees trust leaders, this aids in the development of high-quality exchange relationships (LMX; [162]), which may also encourage employees to spend more (personal) resources and energy on job tasks [163,164].
3. Job and personal resources. In total, nine personal and nine job resources were found to be significant mediators in the relationship between different positive leadership styles and engagement. With regards to job resources, job autonomy and ‘job resources in general’ were most researched (three studies with significant results; see Table 5). Next, the overall congruence of person and job was found to be a mediator twice [80,143]. Only one study found a positive mediating effect of role clarity [1]. With regards to personal resources, only optimism and self-efficacy were found to be significant mediators in two studies, other personal resources were positive effect [147], work–life enrichment [148], project identification [149], practicing core values [150], and psychological capital [151].
  These results are in line with expectations based on the job demands resources model (JD-R model), which posits the importance of personal and job resources for work engagement. Recently, engaging leadership was added to the model [3], indicating that leadership that inspires, connects, and strengthens followers has an indirect, positive effect on their levels engagement through the allocation of job resources and job demands.
4. Organizational and team resources. Seven studies investigated mediators at levels other than the individual employee–leader level. Six of them were organizational-level mediators. Two studies focused on organizational identification [117,140], while two other studies focused on social corporate goals as mediators: i.e., corporate social responsibility [153] and perceived societal impact [154]. Only one study investigated organizational justice [152] and ‘promotive organization-based psychological ownership’ [155]. At the group level, only one study found group identification to be a mediator in the relationship between transformational leadership and engagement [154].
  These results provide evidence for the importance of incorporating multilevel mediators when researching the relationship between positive leadership styles and engagement, specifically organizational identification and social corporate goals.
5. Leader attributes. Two studies found that leadership effectiveness [47] and perceived support [156] were mediators with regards to the relationship of transformational and authentic leadership, respectively.

### 5.3. Summary

**Figure 1:**
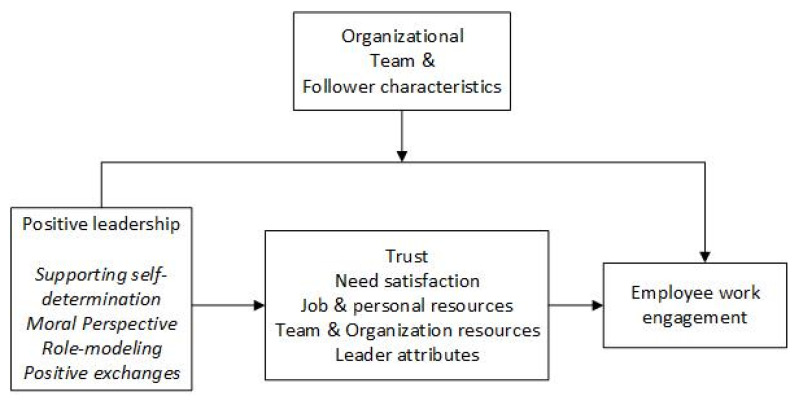
Empirical research model based on the mediating and moderating mechanisms from studies in the meta-analysis. The three behaviours of positive leadership styles in italics are based on a theoretical comparison. The overarching categories over mediators and moderators can be found in the middle squares, in the order of magnitude with regards to the amount of studies in each category. Resources can be further divided into job resources and personal resources.

Our categorization of studies show that a number of moderating and mediating influence the relationship between positive leadership styles and engagement. Psychological variables, i.e., psychological needs, made up the largest category (k = 13). The second largest category included studies concerning trust in the leader and the organization (k = 10). Third, both job resources (k = 9) and personal resources (k = 9) were well-researched mediators. The fourth category consisted of team and organizational resources (k = 7). Last, we found two studies with regards to leader attributes. These categories of variables may be shared mediating mechanisms between positive leadership styles and engagement. In addition, our theoretical analysis as well as the meta-analysis provided evidence for a common ground between all positive leadership styles (see above); therefore, we propose the following overarching research model to guide future research (see Figure 1).

## 6. Discussion

In this study, we set out to empirically investigate the black box of the relationship between positive leadership styles and work engagement. We respond to calls for studies with an integrative view on leadership [33], for an integration across leadership styles [34], and for an investigation of overlap between leadership styles [35] by using both a deductive and an inductive approach, with both quantitative and qualitative analyses. We found shared theoretical mechanisms shared between positive leadership styles, we quantified the positive association between (positive) leadership styles and work engagement through a meta-analysis, and we identified several categories of mediating and moderating mechanisms in an overarching research model that may further explain these associations and guide future research.

The deductive theoretical analysis indicated that transformational, authentic, servant, ethical, and empowering leadership share overlap in their focus on being a moral manager, role modelling behaviour, supporting employee self-determination, and fostering positive exchanges with employees. These shared leader behaviours are in line with a shift in the leadership domain from more inspirational leadership to a more moral leadership framework that seems to rest more heavily on values, morality, empathy, and service [74]. The clear overlap between these positive leadership styles could, in part, also be due to construct mixology, i.e., the practice of building new psychological constructs by combining older constructs [88]. This is not necessarily a bad thing, although construct redundancy among newer positive leadership styles seems to be an issue [74]. In any case, some never positive leadership styles may have been ‘borrowed’ some elements from older research on leadership styles. A second explanation may lie in rather similar communication tactics at a behavioural level; leaders spend most of their time communicating with employees, whether directly or indirectly [165], which builds the leader–employee relationship [36]. In addition, being a moral manager or role modelling prescribes communication about ethics, while supporting self-determination means that a leader has attention for employee autonomy, competence, and relatedness during regular conversations or performance reviews. Lastly, the shared element ‘fostering positive exchanges’ directly indicates the importance of leader communication.

The meta-analysis showed a positive and significant association overall (r = 0.47), as well as for each leadership style separately (from r = 0.34 for servant leadership up to r = 0.52 for ethical leadership). Our population correlations can be qualified as large (r = 0.47; [107]) and are similar to the results from previous meta-analyses with smaller sample sizes and fewer leadership styles [66,73]. Contrary to Hoch et al. [66], we did not find that servant leadership had the highest association with work engagement. However, our findings are similar to what is found in longitudinal research and multisource and experimental research [39,47,83]. We found only one multisource study where the correlation between transformational leadership and employee engagement (r = 0.34) dropped to a non-significant level when the leaders rated their own leadership (r = −0.09; [39]). The moderated meta-analysis with the leadership category as a moderator did not indicate any significant differences between leadership styles. Moreover, the confidence and credibility intervals of each leadership style overlapped. These results indicate that there might indeed be common ground with regards to the effect of different leadership styles on work engagement that can be explained by the shared leader behaviours identified above.

However, significant heterogeneity (see Q-statistic, Table 5) was present within the results of the meta-analysis, indicating the presence of moderating variables in the leadership–engagement relationship. In order to investigate this further, we first conducted a moderated meta-analysis with the engagement questionnaire, the sample origin (western vs. non-western), and industry as moderators, which did not yield any results. In order to further search for trends in explaining mechanisms, we looked at the moderating and mediating variables in the individual studies of the meta-analysis. The moderators in the sample were quite heterogeneous, indicating mostly that various personal and organizational-level moderators influenced the relationship between positive leadership styles and engagement. Of course, leadership does not exist in a vacuum, so we suggest that future research looks into organizational level boundary conditions and uses more multi-level or time-sensitive research approaches to capture the unexplained variance found in our meta-analysis [166].

We did find a clear pattern with regards to mediating mechanisms. The psychological needs category was the most researched category; this is not surprising, since two highly popular engagement theories posit the importance of psychological variables: self-determination theory [63] states that the enhancement of autonomy, relatedness, and competence leads to work engagement, and the theory of Kahn [58] posits that three psychological conditions, i.e., availability, meaningfulness, and safety, influence work engagement. This supports the notion that the employee psychological need of satisfaction is of definite importance to work engagement [64] and that positive leadership styles implicitly or explicitly acknowledge this already in their theoretical framework. Leaders who focus more on employee self-determination and who are spending more time strengthening, connecting and inspiring their followers [3] may have a more beneficial impact on work engagement.

The second most researched mediator category was trust, indicating that the enhancement of employee trust is a vital process through which employee engagement can be augmented. Again, two of the theoretical shared leader mechanisms relate to the enhancement of trust, i.e., being a moral manager and being a role model. This can be explained by a character-based perspective on trust, which implies that followers attempt to draw inferences about the leader’s characteristics (i.e., integrity, fairness, ability, etc.), which then inform work behaviour and employee attitudes. In this view, perceptions about the trustworthiness of leaders become important, since leaders have authority to make decisions that have an impact on the follower and, thus, make them vulnerable [161]. Perceived leader behavioural integrity and perceived transparent communication have indeed been related to employee engagement [167] as have leader procedural and interactional fairness [168]. Leader action and practices, thus, infuse trust in their employees [161]. Being a moral manager and a role model, which enhances employee trust, may, therefore, be important shared leader mechanisms through which positive leadership styles can influence engagement.

The third mediator category concerned personal and job resources. This can be explained by the job demands–job resources model [55], in which it is posited that resources, be it personal or job resources, energize an employee and increase work engagement.

The fourth category with team- and organizational-level resources shows the importance of investigating leadership processes and employee consequences from a wider, organizational perspective. The multilevel leadership field is still emerging and rather fragmented; therefore, calls have been made for a more thorough investigation of leadership phenomena through this research lens [169].

Finally, leader attributes influence the relationship between leadership and engagement, although this category consisted of few studies. It is not hard to imagine that several leader characteristics may influence the quality of the relationship with the leader, and therefore, the level of engagement of the employee. Research has, e.g., shown that leader characteristics, including personality traits, explain the most variance in the exchange relationship [170].

Several of the theoretically deduced shared leader behaviour and empirically researched mediators also seem to be directly associated with each other: being supportive for employee self-determination (shared leader behaviour) influences psychological needs (mediator category), which leads to engagement. Similarly, having a moral perspective and being a role model (shared leader behaviour) can be related to the development of trust (mediator category), which then leads to engagement. The last shared leader behaviour category, positive exchanges with employees, may lead to a different allocation of resources by the leader in favour of the employee. We believe that our research model proposes an integrated framework developed to understand the shared effect of all the positive leadership styles in our review. Some positive leadership styles, however, may focus more on certain pathways than others; e.g., experimental research from Van Dierendonck et al. [47] showed that both transformational and servant leadership were related to work engagement; yet, transformational leaders were perceived as more effective, while servant leaders were better at fulfilling followers’ needs.

We simply propose that some of the underlying mechanisms may be the same. For future research, therefore, we encourage leadership researchers to either (1) control for shared influencing mechanisms (e.g., LMX) when studying effects of a single positive leadership style on, e.g., engagement, or (2) to focus more on common mechanisms and their translation at the behavioural level (e.g., the role of communication behaviour).

### 6.1. Limitations and Future Research

In the meta-analysis and review, only peer-reviewed studies were included to ensure the quality of the research. A possible caveat is the risk of over-representing positive and significant results, although the meta-analysis did not seem to indicate publication bias. Only with the leadership styles with fewer studies (servant and empowering leadership) did the Trimfill analysis add studies to counteract publication bias, but this did not drastically alter the results. Furthermore, the data in the meta-analysis were cross-sectional, so no inferences concerning causality can be made. This also points out the possibility of endogeneity and common source bias [171], because employees in the meta-analysis rated both their leader and their own engagement using self-report questionnaires. However, longitudinal, multisource, and experimental studies show similar results ([39,47,129]). Additionally, for the inductive approaches (both quantitative and qualitative), we were limited to the research that was present. This research may be guided by popular theoretical rationales and, hence, influence the amount of studies that were present with a certain mediating or moderating mechanism. We can only encourage future research to take into account multiple mechanisms and perhaps to test them simultaneously. To this regard, testing and modelling multiple mediation paths will help test the proposed research model [166].

It would be interesting if future research focuses more on similarities between different leadership styles, either theoretically (on a dimensional or definitional level) or empirically; future research can, e.g., focus on further examining overlap between positive leadership styles on a more behavioural level. To accomplish this aim, perhaps diary studies [81], combined with a multilevel approach [172], might be an interesting research avenue. Additionally, the focus on how to build positive relationships with followers has been a research question for a while [170], which is why future research may want to focus more on underlying communication behaviour as a mediator. Lastly, team engagement [173] and engaging leadership [3] are interesting developments in the literature that will extend our understanding of how leadership influences employee engagement.

### 6.2. Practical Implications

Positive leadership styles are significantly and positively related to work engagement. Although each leadership style has its own focus, they do seem share a common ground with regards to their effect on work engagement. Positive leaders seem to provide a moral perspective, act as role models, support follower self-determination (autonomy, competence, and relatedness), and foster positive social exchanges. Focusing on these elements in selection or training of leaders may dramatically increase work engagement. For more practical recommendations or interventions with regards to this topic see [174] (p. 341).

In addition, leaders can also have a positive influence on work engagement through trust enhancement, better resource allocation, and positive organizational level initiatives, all of which serve as pathways through which effects on work engagement manifest. In sum, there are many ways leaders can enhance work engagement. It is well worth the effort, not only because higher work engagement enhances general wellbeing, but—if more convincing is needed—work engagement (and positive emotion) (1) may be contagious and, therefore, enhance general firm wellbeing [67,175] as well as (2) increase employees’ (creative) performance and productivity [14,15,176].

## 7. Conclusions

In sum, when empirically (and inductively) comparing transformational, ethical, servant, authentic, and empowering leadership, we cannot conclude that there is a positive leadership style that is best for work engagement, as the meta-correlations were all in the same order of magnitude. Moreover, since all these positive leadership styles have overlapping credibility and confidence intervals, one can also assume that there are shared processes underneath these leadership styles that influence work engagement. We did not find a meta-moderating influence of the education level of employees, leadership or engagement questionnaire, sample size, or publication year; this indicates that the results are generalizable. Based on deductive theoretical analyses of the underlying leadership theories, we identified several shared behaviours across leadership styles that may explain the relatively high meta-correlations with work engagement, i.e., focusing on employee self-determination, fostering positive social exchanges, moral behaviour, and role modelling. In addition, based on the empirical analysis of the articles within the samples, we propose several categories of mediating and moderating mechanisms that may influence the leadership–work engagement relationship. Moderating categories were: employee-level attributes and team- and organizational-level moderators, whereas mediating categories were psychological needs, trust, personal resources, job resources, organizational resources, leader attributes, and team-level resources. These categories map nicely on the proposed theoretical explanations of the leadership–engagement nexus. The overarching research model resulting from the deductive and inductive analysis in this article may help guide future research, as well as advise HR personnel in organizations with regards to interventions to help increase employee work engagement.

## Figures and Tables

**Table 1:** Positive leadership styles and their components.

Transformational Leadership	Authentic Leadership	Servant Leadership	Ethical Leadership	Empowering Leadership
Idealized influence	Self-awareness	Empowerment	Moral person	Delegation of authority
Intellectual stimulation	Balanced processing	Accountability	Moral manager	Accountability for outcomes
Inspirational motivation	Relational transparency	Standing back		Self-directed decision making
Individualized consideration	Internalized moral perspective	Humility		Information sharing
		Authenticity		Skills development
		Courage		Coaching for innovative performance
		Forgiveness		
		Stewardship		

**Table 2:** Results meta-analysis.

Leadership	*N*	k	r	r_c_	ρ	SE	Q	95% CI	80% CR	R^2^	N_FS_	Trimfill (SE)
Total	37,905	86	0.42 ****	0.47 ****	0.47 ****	0.04	1311.76 ****	(0.40; 0.53)	(0.25; 0.68)	22.09%	254,154	Right: 0 (5.28)
Transformational	23,194	43	0.43 ****	0.47 ****	0.47 ****	0.04	502.13 ****	(0.40; 0.55)	(0.30; 0.64)	22.09%	75,068	Left: 0 (3.89)
Authentic	7656	21	0.39 ****	0.43 ****	0.43 ****	0.07	603.91 ****	(0.30; 0.55)	(0.08; 0.77)	18.49%	10,824	Right: 0 (2.51)
Servant	1806	4	0.34 *	0.39 *	0.31 ***	0.09	79.46 ****	(0.13; 0.49)	(0.19; 0.59)	9.61%	442	Left: 2 (1.47)
Ethical	3681	10	0.52 ****	0.56 ****	0.56 ****	0.03	40.69 ****	(0.51; 0.62)	(0.46; 0.66)	31.36%	6341	Right: 0 (2.12)
Empowering	1568	8	0.38 ****	0.42 ****	0.46 ****	0.04	31.97 ***	(0.39; 0.54)	(0.31; 0.54)	21.16%	846	Right: 3 (1.87)

* *p* < 0.05; *** *p* < 0.001; **** *p* < 0.0001; *N* = number of participants; k = number of studies; r = bare-bones Hunter and Schmidt method using Metafor (corrected for sample size); r_c =_ corrected for attenuation; ρ = corrected for publication bias with Trimfill method; SE = standard error, Q = heterogeneity test; 95% CI = 95% confidence interval; CR = 80% credibility interval; R^2^ = percentage of explained variance; N_FS_ = fail safe N; Trimfill (SE) = number of studies added to account for publication bias at the left or right of the average individual study correlation.

**Table 3:** Shared leadership attributes between different leadership styles based on theoretical comparisons.

Study	Leadership Attributes Based on Theory	Transformational Leadership	Servant Leadership	Authentic Leadership	Ethical Leadership
[109]	Influence	X	X		
	Vision	X	X		
	Trust	X	X		
	Respect or credibility	X	X		
	Risk-sharing or delegation	X	X		
	*Integrity*	X	X		
	*Role modelling*	X	X		
[42]	*Internalized moral perspective* (authentic leadership)	X		X	X
	*Moral person* (ethical leadership)	X		X	X
	Moral manager (ethical leadership)	*x*		*x*	X
	Idealized influence (transformational leadership)	X		*x*	X
[111]	*Positive moral perspective*	X	X	X	
	Leader self-awareness of values, cognitions, and emotions	X	X	X	
	Leader authentic behaviour	*x*	X	X	
	*Positive role modelling*	X	X	X	
	Personal and social identification	X	*x*	X	
	*Supporting self-determination*	X	X	X	
	*Positive social exchanges*	X	*x*	X	
	Follower self-awareness of values	X	X	X	
	Follower internalized self-regulation	X	*x*	X	
[49]	Concern for others (altruism)	X		X	X
	*Ethical* decision making	X		X	X
	Integrity	X		X	X
	*Role modelling*	X		X	X

Between brackets, original theory on which the comparison was based; ‘X’ = focal point in the theory; small ‘*x*’ = discussion of the attribute in a theory; ref. [109] compared transformational and servant leadership; [42,49] compared transformational, authentic, and ethical leadership; ref. [111] compared transformational, servant, and authentic leadership. Explanation of bold: themes that reoccur in each comparison article.

**Table 4:** Moderators of the relationship between positive leadership styles and engagement in empirical research. Between brackets the ‘amount’ of the moderator related to a higher employee work engagement.

Categories	Moderators	Study	Leadership Style
Follower characteristics	(high) Positive follower characteristics (independent thinking, willing to take risks, active learner, innovative)	[121]	Transformational leadership
	(high) Leader–follower social capital (i.e., goal congruence and social interaction)	[122]	Servant leadership
	(high) Promotion focus	[6,116]	Transformational leadership Ethical leadership
	(high) Person–job fit	[123]	Transformational leadership
	(high) Intrinsic motivation	[124]	Authentic leadership
	(high) Need for leadership (moderating effect on need fulfilment, leads to engagement)	[125]	Transformational
	(high) Cognitive emotion regulation	[126]	Ethical leadership
	(high) Ethical ideology (moderating effect on justice perception, which leads to engagement)	[127]	Ethical leadership
	(high) Self-efficacy	[117,128]	Servant leadership Empowering leadership
Organizational	(high) Uncertainty		
characteristics	(less) Beneficiary contact	[119]	Authentic leadership
	(more) Supportive culture	[118]	Transformational
Team characteristics	(low) Group job satisfaction	[120]	Ethical leadership

**Table 5:** Mediators in the leadership–engagement relationship from articles in the meta-analysis.

Categories	Mediator	Study	Leadership Style
Psychological needs	Competence need satisfaction	[129]	Transformational
	Relatedness need satisfaction	[129]	Transformational
	Psychological need satisfaction	[47]	Servant
	Need satisfaction	[125]	Transformational
	Meaningfulness	[130]	Transformational
	Perceptions of meaning in work	[131]	Transformational
	Work meaningfulness	[132]	Empowering
	Meaningfulness	[126]	Ethical
	Psychological empowerment	[1,51,53,117,133]	Servant Empowering (3x) Authentic
Trust	(employee) Trust (in leader)	[70]	Ethical
		[134]	Ethical
		[19]	Ethical
		[135]	Authentic
		[71]	Authentic
		[136]	Authentic
		[137]	Authentic
	Trust in organization	[72]	Authentic
	Trust climate (organizational)	[138]	Servant
	Interpersonal trust in leader (i.e., leader’s competence, leader’s benevolence, leader’s reliability)	[139]	Authentic
Job resources	Job autonomy	[140]	Transformational
		[141]	Transformational
	(not significant)	[129]	Transformational
	Responsibility	[130]	Transformational
	Role clarity	[1]	Empowering
	Job resources in general	[125]	Transformational
		[142]	Transformational
	Overall person–job match	[80]	Authentic
	Person–job Fit	[143]	Transformational
Personal resources	Self-efficacy	[144]	Transformational
	Self-efficacy	[145]	Transformational
	Optimism	[72]	Authentic
	Academic optimism	[146]	Authentic
	Positive affect	[147]	Transformational
	Work–life enrichment	[148]	Authentic
	Project identification	[149]	Transformational
	Practicing core values	[150]	Authentic
	Psychological capital	[151]	Empowering
Organizational and team resources	Organizational identification	[117]	Servant
		[141]	Transformational
	Organizational justice	[152]	Ethical
	Corporate social responsibility	[153]	Transformational
	Perceived societal impact	[154]	Transformational
	Promotive organization-based psychological ownership	[155]	Authentic
	Group identification	[154]	Transformational
Leader Attributes	Leadership effectiveness	[47]	Transformational
	Perceived support	[156]	Authentic

## Data Availability

All data can be found in the Appendix A.

## Appendix A

**Table A1:** Study information from meta-analysis sample.

Author (Year)	Leadership Style	Leadership Measure	Alpha	Engagement Measure	Alpha	Country	N	Industry	r
Abidin (2017) [37]	Authentic	ALQ 16 (Walumbwa et al., 2008)	0.76	WES 18 (Rich et al., 2010)	0.88	Malaysia	260	Budget hotels	0.32
Adil and Kamal (2016) [177]	Authentic	ALQ 16 (Walumbwa et al., 2008)	0.93	UWES 9 (Schaufeli et al., 2006)	0.95	Pakistan	500	University teachers	0.29
Albrecht and Andreetta (2011) [53]	Empowering	Empowering subscale (Pearce and Sims, 2002)	0.91	UWES 9 (Schaufeli et al., 2006)	0.87	Australia	139	Community health service	0.34
Alok and Israel (2012) [155]	Authentic	ALQ 16 (Walumbwa et al., 2008)	0.95	UWES 9 (Schaufeli et al., 2006)	0.88	India	117	Working professionals	0.47
Arfat et al. (2017) [119]	Transformational	MLQ 5x (Bass and Avolio, 1995)	0.81	Engagement (Saks, 2006)	0.83	Pakistan	700	Banking	0.58
Azanza et al. (2015) [178]	Authentic	ALQ (Walumbwa et al., 2008)	0.89	UWES 9 (Schaufeli et al., 2006)	0.89	Spain	623	Various	0.54
Bae et al. (2013) [179]	Transformational	MLQ 12 (Bass and Avolio, 1992)	0.97	UWES 9 (Schaufeli and Bakker, 2003)	0.92	US	304	School teachers	0.34
Bass et al. (2016) [180]	Transformational	4 items (adapted from Pearce and Sims, 2002)	0.91	UWES 9 (Schaufeli et al., 2006)	0.84	US	728	School employees	0.28
Besieux et al. (2015) [153]	Transformational	MLQ 13 items (Avolio et al., 1999)	0.95	18 items (Towers Watson, 2010)	0.86	Belgium	5313	Banking	0.48
Bird et al. (2012) [181]	Authentic	ALQ (Walumbwa et al., 2008)	0.84	Q12 Gallup (Buckingham and Coffham, 1999)	0.88	US	633	Teaching staff	0.61
Breevaart et al. (2014) [126]	Transformational	TLI (Podsakoff et al., 1990)	0.91	UWES 9 (Schaufeli et al., 2006)	0.94	Netherlands	162	Various	0.53
Bui et al. (2017) [145]	Transformational	MLQ 20 (Avolio and Bass, 2004)	0.97	UWES 9 (Schaufeli et al., 2002)	0.96	China	691	Various	0.64
Buil et al. (2016) [182]	Transformational	7 items (Carless et al., 2000)	0.90	UWES 9 (Schaufeli et al., 2003)	0.89	Spain	323	Receptionists in hotels	0.51
Cerne et al. (2014) [85]	Authentic	ALI 16 (Neider and Schriesheim, 2011)	0.94	UWES 9 (Schaufeli et al., 2006)	0.75	Slovenia	171	Manufacturing and processing	0.32
Cheng et al. (2014) [117]	Ethical	ELS 10 items (adapted Brown et al., 2005)	0.93	WES 18 items (Rich et al., 2010)	0.96	Taiwan	670	Economic research	0.48
De Clercq et al. (2014) [123]	Servant	SLQ 28 items (Liden et al., 2008)	0.96	UWES 17 (Schaufeli et al., 2006)	0.90	Ukraine	263	IT companies	0.50
De Klerk and Stander (2014) [51]	Empowering	LEBQ (Konczak et al., 2000)	0.91	WES (Rothmann, 2010)	0.90	SA	322	Various production areas	0.36
Demirtas (2015) [127]	Ethical	ELS 10 (Brown et al., 2005)	0.95	UWES 17 (Schaufeli et al., 2002)	0.88	Turkey	418	Firm in aviation logistics	0.49
Demirtas (2017) [128]	Ethical	ELS 10 (Brown et al., 2005)	0.93	UWES 9 (Schaufeli et al., 2002)	0.92	US	317	Aviation maintenance	0.48
Den Hartog and Belschak (2012) [183]	Ethical	ELS 10 (Brown et al., 2005)	0.91	UWES 9 (Schaufeli and Bakker, 2004)	0.92	Netherlands	167	Various jobs	0.54
Den Hartog and Belschak (2012) [183]	Ethical	ELS 10 (Brown et al., 2005)	0.88	UWES 9 (Schaufeli and Bakker, 2004)	0.91	Netherlands	200	Various jobs	0.49
Ding et al. (2017) [151]	Transformational	MLQ 5x (Bass and Avolio, 1994)	0.94	WES 9 (Rich et al., 2010; He et al., 2014)	0.90	China	162	Infrastructure projects	0.27
Engelbrecht et al. (2014) [70]	Ethical	LES 17 (this study)	0.97	UWES 17 (Schaufeli and Bakker, 2003)	0.89	South Africa	204	Various orgs	0.60
Enwereuzor et al. (2016) [124]	Transformational	TLI 22 (Podsakoff et al., 1990)	0.83	UWES 9 (Schaufeli et al., 2006)	0.89	Nigeria	224	Hospital nurses	0.50
Espinoza-Parra et al. (2015) [184]	Transformational	MLQ 5x short (Molero et al., 2010)	0.95	UWES 17 (Salanova et al., 2000)	0.90	Chile	985	Police officers	0.45
Ghadi et al. (2013) [132]	Transformational	GTL (Carless et al., 2000)	0.95	UWES 9 (Bakker, 2009)	0.95	Australia	530	Various	0.69
Giallonardo et al. (2010) [185]	Authentic	ALQ 16 (Avolio et al., 2007)	0.91	UWES 17 (Schaufeli and Bakker, 2003)	0.86	Canada	170	Registered nurses	0.21
Goswami et al. (2016) [186]	Transformational	TLQ 24i (Podsakoff et al., 1990)	0.87	UWES 17 (Schaufeli et al., 2006)	0.93	US	235	Consulting	0.08
Gözükara and Simsek (2015) [141]	Transformational	MLQ 5x (Bass and Avolio, 1995)	0.96	UWES 17 (Schaufeli and Bakker, 2003)	0.90	Turkey	101	Academic staff	0.34
Gözükara and Simsek (2016) [142]	Transformational	MLQ 5x (Bass and Avolio, 1995)	0.96	UWES 17 (Schaufeli and Bakker, 2003)	0.90	Turkey	252	Higher education	0.47
Hansen et al. (2014) [79]	Transformational	TFL 15 (Rafferty and Griffin, 2004)	0.96	UWES 9 items (Schaufeli et al., 2002)	0.92	US	451	International firm	0.42
Hassan and Ahmed (2011) [140]	Authentic	ALQ 19 items (Avolio et al., 2007)	0.91	UWES 9 (Schaufeli and Bakker, 2004)	0.91	Malaysia	395	Banking	0.41
Hawkes et al. (2017) [143]	Transformational	LBS (Podsakoff et al., 1990)	0.96	UWES 9 (Schaufeli et al., 2006)	0.93	Australia	277	Various	0.47
Hayati et al. (2014) [187]	Transformational	MLQ 20 (Bass and Avolio, 1997)	0.91	UWES 17 (Schaufeli et al., 2002)	0.73	Iran	240	Nurses	0.70
Hsieh and Wang (2015) [137]	Authentic	ALQ (Walumbwa et al., 2008)	0.88	UWES 17 (Schaufeli et al., 2002)	0.95	Taiwan	345	Manufacturing and service	0.61
Jiang and Men (2017) [149]	Authentic	Neider and Schriesheim (2011)	0.97	11 items (Kang 2014; Saks, 2006)	0.96	US	391	Various	0.55
Joo et al. (2016) [188]	Authentic	ALQ (Avolio et al., 2005)	0.88	UWES 9 (Schaufeli et al., 2006)	0.91	Korea	599	Knowledge workers	0.47
Khuong and Dung (2015) [135]	Ethical	ELS 10 (Brown et al., 2005)	0.93	UWES 17 (Schaufeli et al., 2002)	0.90	Vietnam	312	Technicians	0.37
Khuong and Yen (2014) [189]	Ethical	ELS 10 (Brown et al., 2005)	0.93	UWES 17 (Schaufeli et al., 2002)	0.92	Vietnam	269	5 industries	0.48
Kulophas et al. (2018) [147]	Authentic	ALQ (Walumbwa et al., 2008)	0.92	UWES 18 (Schaufeli et al., 2006)	0.93	Thailand	605	Teachers several schools	0.36
Kopperud et al. (2014) [39]	Transformational	MLQ 20 (Bass and Avolio, 1990)	0.82	UWES 9 (Schaufeli et al., 2006)	0.92	Norway	1226	Financial services	0.44
Kopperud et al. (2014) [39]	Transformational	MLQ 20 (Bass and Avolio, 1990)	0.91	UWES 9 (Schaufeli et al., 2006)	0.89	Norway	291	Audit company	0.34
Kovjanic et al. (2013) [130]	Transformational	MLQ 19 (Bass and Avolio, 1995)	0.97	UWES 9 (Schaufeli et al., 2006)	0.95	Germany	190	Various	0.71
Lee et al. (2017) [133]	Empowering	LBQ (Pearce and Sims, 2002)	0.86	UWES 9 (Schaufeli et al., 2006)	0.91	Malaysia	134	Various	0.37
Lewis and Cunningham (2016) [190]	Transformational	TFL 18 (Rafferty and Griffin, 2004)	0.97	UWES 17 (Schaufeli and Bakker, 2003)	0.88	US	120	Nurses	0.46
Manning (2016) [191]	Transformational	MLQ 5x (Bass and Avolio, 1995)	0.91	UWES 17 (Schaufeli and Bakker, 2003)	0.90	US	441	Staff nurses, 3 hospitals	0.37
Mauno et al. (2016) [192]	Transformational	GTL 7 items (Carless et al., 2000)	0.94	UWES 9 (Schaufeli et al., 2006)	0.93	Finland	3466	Nurses	0.29
Mayr (2017) [155]	Transformational	MLQ 20 (Bass and Avolio, 1995)	0.96	UWES 9 (Schaufeli et al., 2006)	0.93	Germany	213	Volunteer fire fighters	0.40
Mendes and Stander (2011) [1]	Empowering	LEBQ (Konczak et al., 2000) + 2 items info sharing (Arnold et al., 2000)	0.88	UWES 9 (Schaufeli et al., 2002)	0.83	South Africa	179	Chemical org	0.25
Mitonga-Monga et al. (2016) [193]	Ethical leadership	ELS 10 (Brown et al., 2005)	0.91	UWES 9 (Schaufeli et al., 2002)	0.90	SA	839	Railway transportation	0.59
Moss (2009) [6]	Transformational	TFL 15 (Rafferty and Griffin, 2004)	0.89	UWES 9 vigor and dedication	0.87	Australia	160	Various	0.35
Mozammel and Haan (2016) [194]	Transformational	MLQ 20 (Avolio and Bass, 2004)	0.91	UWES 9 (Bakker and Schaufeli, 2003)	0.89	Bangladesh	128	Banking	0.18
Ochalski (2016) [195]	Transformational	MLQ 5x (Avolio and Bass, 2004)	0.91	UWES 17 (Bakker, 2011)	0.90	US	157	Pharmaceutical	0.67
Oh et al. (2018) [151]	Authentic	ALQ 16 (Walumbwa et al., 2008)	0.75	UWES 9 (Schaufeli et al., 2006)	0.80	South Korea	281	3 big corporations	0.47
Park et al. (2017) [152]	Empowering	12 items (Ahearne et al., 2005)	0.93	WES 18 (Rich et al., 2010)	0.97	South Korea	285	8 large firms	0.59
Perko et al. (2016) [4]	Authentic	ALQ 16 (Walumbwa et al., 2008)	0.94	UWES 9 vigor (Schaufeli et al., 2006)	0.87	Finland	262	Various, public sector	0.31
Popli and Rizvi (2015) [102]	Transformational	MLQ 5x (Bass and Avolio, 1995)	0.93	DDI E3 (Phelps, 2009)	0.90	India	106	Service sector	0.59
Popli and Rizvi (2016) [196]	Transformational	MLQ 5x (Bass and Avolio, 1995)	0.90	DDI E3 (Phelps, 2009)	0.90	India	329	Service sector	0.42
Prochazka et al. (2017) [146]	Transformational	CLQ (Prochazka et al., 2016) based on MLQ	0.96	UWES 9 (Schaufeli, 2015)	0.92	Czech Republic	307	Various	0.44
Pourbarkhordari et al. (2016) [197]	Transformational	16 items (Wang and Howell, 2010)	0.94	UWES 9 (Schaufeli et al., 2006)	0.81	China	202	Telecommunications	0.40
Qin et al. (2014) [121]	Ethical leadership	ELS 10 (Brown et al., 2005)	0.95	UWES 9 (Schaufeli et al., 2006)	0.90	China	285	Tourism	0.65
Sahu et al. (2018) [104]	Transformational	MLQ 12 (Bass and Avolio, 1992)	0.96	Gallup 12 (Mann and Ryan, 2014)	0.88	India	405	IT	0.54
Salanova et al. (2011) [145]	Transformational	MLQ 20 (Bass and Avolio, 1997)	0.78	UWES 17 vigor and dedication (Schaufeli et al., 2002)	0.84	Portugal	280	Nurses	0.18
Scheepers and Elstob (2016) [120]	Authentic	ALQ (Avolio et al., 2007)	0.90	UWES 9 (Schaufeli et al., 2006)	0.54	South Africa	81	Financial service orgs	0.52
Schmitt et al. (2016) [198]	Transformational	11 items Dutch scale (De Hoogh et al., 2004)	0.94	UWES 9 (Schaufeli et al., 2002)	0.91	Netherlands	148	Various	0.37
Seco and Lopes (2013) [199]	Authentic	ALQ (Walumbwa et al., 2008)	0.89	UWES 9 (Schaufeli et al., 2006)	0.94	Portugal	326	Teachers several schools	−0.57
Shu et al. (2015) [125]	Authentic	ALI 16 (Neider and Schriesheim, 2011)	0.87	UWES 9 (Schaufeli et al., 2006)	0.91	Taiwan	350	Chinese workers	0.18
Song et al. (2013) [200]	Transformational	MLQ 12 (Bass and Avolio, 1992)	0.91	UWES 9 (Schaufeli et al., 2006)	0.95	US	284	CTE teachers	0.34
Song et al. (2012) [201]	Transformational	MLQ 6x 12 (Bass and Avolio, 1992)	0.85	UWES 9 (Schaufeli et al., 2006)	0.74	Korea	432	6 for-profit orgs	0.38
Sousa and van Dierendonck (2014) [118]	Servant	SLS 30 items (van Dierendonck and Nuijten, 2011)	0.79	UWES 9 (Schaufeli et al., 2002)	0.90	Portugal	1107	Two merging companies	0.22
Sousa and van Dierendonck (2017) [202]	Servant	SLS 30 items (van Dierendonck and Nuijten, 2011)	0.93	UWES 9 (Schaufeli et al., 2002)	0.94	Portugal	236	Various	0.55
Stander et al. (2015) [72]	Authentic	ALI (Neider and Schriesheim, 2011)	0.93	UWES 8 items (Schaufeli et al., 2002)	0.90	South Africa	633	27 Hospitals	0.42
Strom et al. (2014) [16]	Transformational	MLQ 20 (Bass and Avolio, 1990)	0.97	UWES 9 (Schaufeli et al., 2006)	0.96	US	348	Various	0.44
Tims et al. (2011) [203]	Transformational	MLQ 12 (Bass and Avolio, 1990)	0.85	UWES 9 (Schaufeli et al., 2006)	0.89	Netherlands	42	Various, 2 different orgs	0.35
van Dierendonck et al. (2014) [47]	Servant	SL 14 (Ehrhardt, 2004)	0.93	UWES 9 (Schaufeli et al., 2006)	0.94	Netherlands	200	Support staff university	0.49
van Schalkwyk et al. (2010) [204]	Empowering	LEBQ (Konczak et al., 2000)	0.96	UWES 17 (Schaufeli et al., 2002)	0.93	South Africa	168	Petrochemical lab	0.39
Vincent-Höper et al. (2012) [205]	Transformational	MLQ 5x, 20 items (Bass and Avolio, 1995)	0.97	UWES 9 (Schaufeli et al., 2006)	0.95	Germany	1132	Various	0.46
Wang and Hsieh (2013) [71]	Authentic	ALQ (Walumbwa et al., 2008)	0.94	UWES 17 (Schaufeli et al., 2002)	0.95	Taiwan	386	Manufacturing and service	0.58
Wang et al. (2017) [148]	Transformational	TLI 22 (Podsakoff et al., 1990)	0.92	UWES 17 (Schaufeli et al., 2002)	0.90	China	422	IT company	0.47
Wefald et al. (2011) [206]	Transformational	GTL 7 items (Carless et al., 2000)	0.95	UWES 9 (Schaufeli et al., 2002)	0.93	Netherlands	382	Finances	0.27
Wei et al. (2016) [207]	Authentic	ALQ (Walumbwa et al., 2008)	0.92	UWES (Schaufeli and Bakker, 2004)	0.92	China	248	Not specified	0.35
Wihuda et al. (2017) [208]	Empowering	12 items (Ahearne et al., 2005)	0.94	UWES 17 (Schaufeli and Bakker, 2004)	0.96	Indonesia	121	Hotels	0.29
Whitford and Moss (2009) [209]	Transformational	TFL 15 vision and recognition (Rafferty and Griffin, 2004)	0.91	UWES 17 vigor and dedication (Schaufeli et al., 2002)	0.89	Australia	165	Various	0.22
Wong et al. (2010) [138]	Authentic	ALQ (Avolio et al., 2007)	0.97	UWES 9 (Schaufeli et al., 2006)	0.90	Canada	280	Registered nurses	0.28
Zhou et al. (2018) [129]	Empowering	10 items (Pearce and Sims, 2002)	0.84	UWES 9 (Schaufeli et al., 2006)	0.81	China	220	11 hotels	0.33
Zhu et al. (2009) [122]	Transformational	MLQ 5x (Bass and Avolio, 1997)	0.84	GWA 12 items (Harter et al., 2002)	0.86	South Africa	140	Various	0.58

**Table A2:** Substitution for Cronbach’s alpha of the engagement questionnaires.

Questionnaire	Average Alpha	Times Substituted
UWES 9 items (Schaufeli et al., 2006)	0.89	4×
UWES 17 items (Schaufeli et al., 2002)	0.90	6×

**Table A3:** Substitution for Cronbach’s alpha of the leadership questionnaires.

Leadership Questionnaire	Average Alpha	Times Substituted
*Transformational leadership*		
GTL (Carless et al., 2000)	0.95	1×
MLQ 20 (Bass and Avolio, 1995)	0.91	5×
*Authentic leadership*		
ALQ (Walumbwa et al., 2008)	0.89	1×
*Ethical leadership*		
ELS (Brown et al., 2005)	0.93	1×

Explanation of bold: these are the leadership styles for which we calculated an average alpha.
